# Predictive Utility of ViroFind Detection of Blood and CSF Virome for Viral Presence in Human Brain Tissue

**DOI:** 10.3390/ijms27062789

**Published:** 2026-03-19

**Authors:** Xin Dang, Barbara A. Hanson, Melissa Lopez, Janet Miller, Millenia Jimenez, Igor J. Koralnik

**Affiliations:** 1 Davee Department of Neurology, Northwestern University Feinberg School of Medicine, Chicago, IL 60611, USA; xin.dang@northwestern.edu (X.D.); barbara.hanson@northwestern.edu (B.A.H.); melissa.lopez@northwestern.edu (M.L.); milleniajimenez@gmail.com (M.J.); 2 Northwestern Medicine, Chicago, IL 60611, USA; jmiller@nm.org

**Keywords:** viral surveillance, blood–brain concordance, predictive utility, metagenomic sequencing

## Abstract

Viral presence in the brain may contribute to chronic neurologic diseases. However, investigating these associations is limited by the difficulty of directly sampling brain tissue in living individuals. Here, we evaluated whether peripheral viral detection using unbiased target-enrichment next-generation sequencing could inform viral presence in the brain across a diverse set of viral taxa. We applied ViroFind to matched brain, blood (peripheral blood mononuclear cells, spleen, and/or lymph node), and cerebrospinal fluid (CSF) to assess the predictive utility of viral detection in blood and CSF for identifying viral presence in brain samples obtained from the National NeuroAIDS Tissue Consortium, including both HIV-infected (HIV^+^) and HIV-uninfected (HIV^−^) individuals without known active viral infection of the brain. Blood negativity was generally more informative for predicting the absence of viruses in the brain than blood positivity for predicting viral presence. CSF viral detection demonstrated limited predictive utility for brain presence across most viral taxa examined. Among blood^+^ individuals, viral burden differed significantly between brain^+^ and brain^−^ cases for Epstein–Barr virus (EBV), parvovirus, and torque teno virus (TTV). Blood viral burden showed moderate ability to distinguish brain^+^ from brain^−^ cases for EBV and parvovirus, and strong discriminatory ability for TTV, with similar decision thresholds across HIV^+^ and HIV^−^ individuals.

## 1. Introduction

Recent advances in metagenomic sequencing-based viral diagnostics have expanded the use of unbiased approaches for viral detection in clinical settings, particularly for suspected encephalitis, meningitis, unexplained neuroinflammatory syndromes, and infections in immunocompromised hosts [1,2,3]. Metagenomic next-generation sequencing (mNGS) of cerebrospinal fluid (CSF) is now used clinically at select centers and reference laboratories as a hypothesis-free diagnostic tool, most prominently in cases where conventional pathogen testing is unrevealing in acute illness [1,4]. In parallel, targeted viral enrichment and hybrid-capture sequencing platforms have been developed to improve sensitivity for viral detection across blood and CSF and are increasingly proposed as scalable approaches for broad viral surveillance in both translational and clinical research contexts [5].

Beyond acute encephalitic presentations, an open question in the field is whether viral detection in accessible compartments such as blood or CSF can serve as a meaningful proxy for viral presence within brain tissue itself, particularly for chronic or low-level infections that may contribute to neurodegenerative disease but fall below the detection paradigms optimized for fulminant central nervous system (CNS) infection. The human brain is an important viral reservoir that is difficult to study directly in vivo. Sampling constraints limit the ability to identify patients with viral infections of the brain directly in living patients; therefore, methods that accurately estimate the likelihood of viral infections of the brain using easily obtained sample types may inform clinical decision-making regarding invasive testing on a case-by-case basis. We previously reported that among 21 viruses identified in peripheral blood-associated samples (Peripheral Blood Mononucleocytes [PBMC], spleen, and lymph node), 15 (71.4%) were also detected in matched brain parenchymal samples [6]. Several of the remaining six viral species identified in the periphery but not in the brain are well known to exhibit neurotropism, including Varicella-Zoster Virus (VZV) and Human herpesvirus 8 (HHV8), yet were never identified in brain tissue samples [7,8]. Conversely, Hepatitis C Virus (HCV), which is not classically considered to be neurotropic, was detected in the periphery of all patients who had the virus present in the brain tissue. Thus, our data suggests that peripheral viral detection and established neurotropic classification do not map uniformly onto viral presence in the brain, motivating a direct, data-driven evaluation of how blood and CSF viral detection relate to viral detection in brain tissue on a viral taxa basis [9]. The study cohort included both HIV-infected (HIV^+^) and HIV-uninfected (HIV^−^) individuals, allowing evaluation of peripheral–CNS viral relationships across differing immune contexts. Because HIV infection is associated with altered immune surveillance and increased susceptibility to viral neuroinvasion, inclusion of both groups provides an opportunity to examine whether concordance between peripheral and brain viral detection is consistent or context-dependent.

Given the diversity of viruses capable of CNS involvement, the critical diagnostic challenge is determining when peripheral viral testing can meaningfully inform the need for invasive evaluation for brain infection. We therefore evaluated the predictive performance of unbiased viral detection in blood and CSF by estimating positive and negative predictive value (PPV/NPV), sensitivity, and specificity for brain viral detection in patients with and without HIV, where sample availability permitted.

Here, we hypothesized that mNGS viral detection in peripheral compartments, through blood and CSF samples, would show virus-specific rule-out or rule-in utility for estimating viral presence in brain tissue. Consistent with this hypothesis, our analyses demonstrate that the absence of viral detection in blood was more informative for predicting the absence of virus in the brain than peripheral positivity for predicting viral presence. In contrast, CSF viral detection provided limited predictive value for the presence of virus in the brain across most viral taxa. In contrast, for a subset of viruses, higher peripheral viral burden was associated with brain involvement, with significant differences observed between brain-positive and brain-negative cases for Epstein–Barr virus (EBV), parvovirus, and torque teno virus (TTV). Quantitative analyses further showed that blood viral burden enabled moderate discrimination of brain involvement for EBV and parvovirus, and strong discrimination for TTV, with comparable operating thresholds across HIV-infected and uninfected individuals.

## 2. Results

ViroFind analysis was performed on 72 matched brain (frontal cortex) and blood-associated samples (PBMC, spleen, and/or lymph node; collectively referred to as “blood”) obtained from the same donors recruited by the NNTC. Among these donors, 24 had matched CSF samples available for analysis. An initial brain-only virome analysis of this cohort, including demographic characteristics and recruitment criteria, has been previously reported [9].

Participants were originally recruited by the NNTC on the basis of HIV status and substance use disorder (SUD), forming four research groups (HIV^+^/SUD^+^, HIV^+^/SUD^−^, HIV^−^/SUD^+^, HIV^−^/SUD^−^). In the present study, SUD status was not included as an analytical factor, as prior brain-only analyses demonstrated minimal differences in overall brain virome composition between SUD^+^ and SUD^−^ individuals, which showed no significant differences in overall viral burden but did show increased HCV prevalence. Accordingly, analyses here focus on compartmental predictive performance rather than prevalence differences by SUD status.

Because CSF samples were disproportionately available from HIV^+^ donors, with limited representation of HIV^−^ individuals, analyses comparing HIV status were not performed for the CSF compartment. Unless otherwise specified, predictive performance metrics reported below reflect pooled analyses across all donors, without stratification by HIV status. Because PPV and NPV are influenced by underlying prevalence, these metrics are interpreted here as cohort-specific estimates, which require expanded population studies before predictive generalizable diagnostic thresholds may be considered.

PPV, specificity, NPV and sensitivity calculations for predictive value of blood samples to correctly identify infections in the brain are shown in Table 1. Further information about identified viruses, including Baltimore classification, genus, full name and abbreviation can be found in Supplemental Table S1. Predictive values were analyzed for their value as a rule-in and rule-out utility. Rule-in utility was defined as a virus with high PPV and specificity with the following ranges: ≥0.9 high, 0.8–<0.9 moderate, and <0.8 poor. Rule-out utility was defined as an NPV and sensitivity ≥ 0.9 high, 0. 8–<0.9 moderate, and <0.8 poor. Consistent with standard diagnostic conventions, rule-in utility emphasizes high specificity and PPV to minimize false positives, whereas rule-out utility emphasizes high sensitivity and NPV to minimize false negatives. Viruses with cohort prevalence < 10% were not annotated for predictive utility.

Rule-out utility was highest for EBV, HHV7, HIV, and HCV. These viruses showed very low rates of false negativity and high rates of sensitivity, indicating that detection in the brain was almost always matched with detection in the blood. This pattern was particularly strong for HCV, for which no instances of brain detection occurred in the absence of blood detection, indicating that peripheral HCV negativity was highly predictive of CNS negativity in this cohort despite HCV not being classically considered neurotropic.

Notably, two of the four viruses demonstrating rule-out utility were herpesviruses, which are highly prevalent but do not typically exhibit sustained peripheral viremia. Because standard clinical assays are optimized to detect acute infection or brief reactivation events, peripheral detection of these viruses is uncommon outside those windows. In contrast, sequencing-based assays detect low-level or intermittent viral material across the genome, likely accounting for the higher peripheral detection rates observed here and indicating that sequencing-based blood testing may be more informative of CNS viral status for these viruses than conventional clinical assays.

To determine whether CSF provided superior predictive performance compared with blood, we next evaluated the predictive utility of viral detection in CSF for identifying brain infection (Table 2).

CSF is commonly considered the most directly accessible compartment for evaluating brain infection; however, CSF viral detection showed limited predictive utility for brain viral presence across most viral taxa examined. JCV, for which virorachia is already an established biomarker for progressive multifocal leukoencephalopathy (PML), was found to have high rule-in utility. Otherwise, CSF testing demonstrated low sensitivity and negative predictive value, indicating that the absence of viral detection in CSF did not reliably exclude viral presence in brain tissue in this cohort. Notably, HBV was the exception where CSF detection exhibited perfect sensitivity and negative predictive value, indicating that brain involvement was not observed in the absence of CSF detection.

Together, these findings indicate that while CSF viral detection can be informative for specific viruses, it was not consistently reliable as a surveillance compartment for estimating brain viral presence in the absence of overt neurological disease, and that its utility varies substantially by viral taxa.

Because immune status is a major determinant of viral persistence and reactivation, we next stratified blood-based predictive analyses by HIV status to determine whether blood–brain viral relationships were stable across immune contexts (Table 3 and Table 4). CSF-based stratification was not performed due to the limited availability of CSF samples from HIV^−^ individuals.

Among viruses with sufficient prevalence in both HIV^+^ and HIV^−^ individuals, only EBV demonstrated robust rule-out utility in both groups, with the absence in blood reliably excluding brain detection regardless of HIV status. HBV was found to have a rule-in utility in HIV^+^ individuals, but did not have sufficient prevalence in HIV^−^ individuals to be analyzed. In HIV^−^ individuals, Sphinx1.76, a replication-competent episomal DNA, and AAV were found to have a rule-in utility when detected in the blood, which was not maintained in the immune-compromised HIV^+^ group. Rule-out utility was strong for HIV^+^ individuals for TTV, HIV, HCV, and HHV7, which was not found in HIV^−^ individuals. HHV6 A/B showed the opposite pattern, meeting rule-out criteria only in HIV^−^ individuals. Together, these findings indicate that while some blood–brain viral relationships are stable across immune contexts, others are strongly modified by host phenotype.

We next evaluated whether blood viral burden, as estimated by viral reads per million (rPM), was associated with viral presence in the brain. Because rPM is a semi-quantitative measure influenced by sequencing depth, library composition, capture efficiency, and viral life stage, it was used here as a relative index of blood viral abundance, rather than a direct measure of viral titer.

Blood rPM were compared between TP and FP cases among individuals with detectable virus in blood, to determine whether blood viral burden differed in association with brain involvement. Viruses with sufficient prevalence for this analysis included HCV, EBV, CMV, parvovirus, HIV, Sphinx, and TTV.

**Figure 1:**
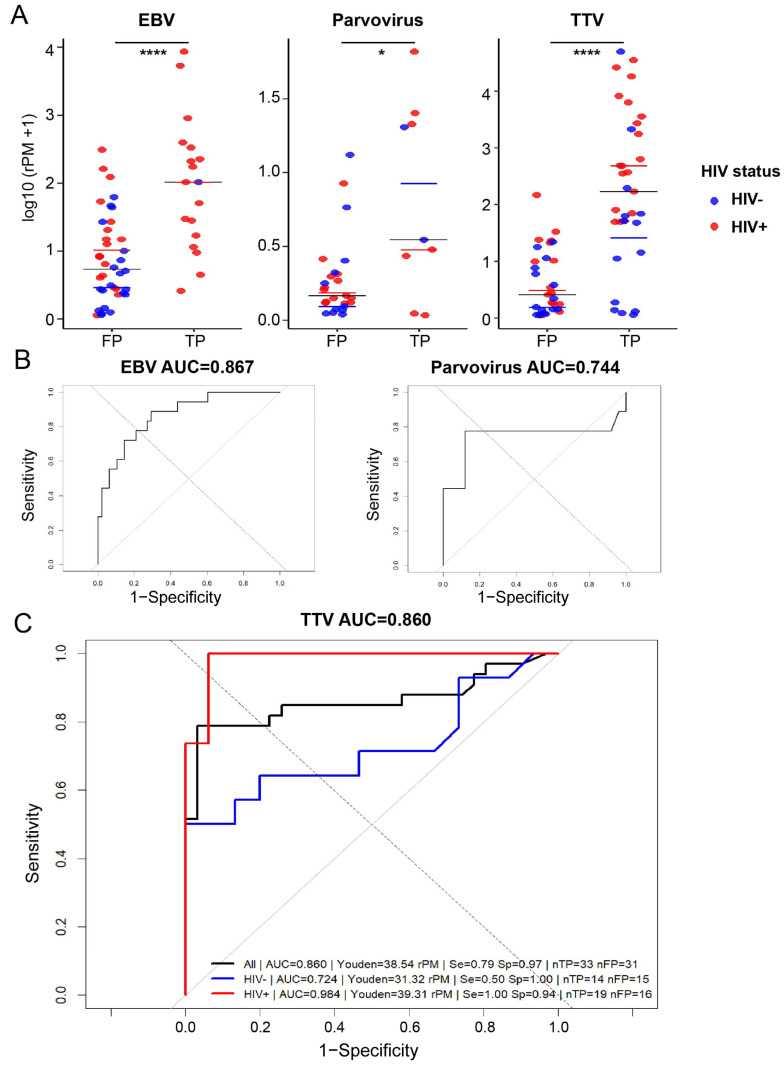
Predictive utility of blood viral burden. (**A**) Scatter plots for viruses with significant Wilcoxon differences in blood viral reads per million (rPM) between false positive (FP; detected in blood but not in brain) and true positive (TP; detected in both blood and brain) cases. Individual samples are shown as points, with HIV^−^ samples indicated in blue and HIV^+^ samples in red. Overall medians are indicated by black horizontal lines. (**B**) Receiver operating characteristic (ROC) curves for EBV and parvovirus (unstratified) and (**C**) TTV showing the combined cohort (black) and stratified HIV^−^ (blue) and HIV^+^ (red) analyses. ROC plots display sensitivity versus 1—specificity, diagonal reference lines indicate performance expected by chance (solid and dashed diagonals indicating no-discrimination and equal sensitivity/specificity, respectively). ROC curves deviating toward the upper-left region reflect improved discriminative ability, with operating thresholds selected using the Youden index to balance sensitivity and specificity. AUC: area under curve, rPM: reads per million; Se: sensitivity; Sp: specificity; nTP: number true positive; nFP: number false positive, *: *p* < 0.05, ****: *p* < 0.0001.

While binary detection of TTV in blood did not provide a reliable rule-in or rule-out utility, quantitative blood viral burden showed strong discriminatory value for identifying brain involvement. Median blood rPM were significantly higher in TP compared with FP cases for TTV (TP: n = 33, median = 166.31 rPM; FP: n = 31, median = 1.57 rPM; *p* < 0.0001), EBV (TP: n = 18, median = 101.81 rPM; FP: n = 38, median = 4.36 rPM; *p* < 0.0001), and parvovirus (TP: n = 9, median = 2.49 rPM; FP: n = 25, median = 0.46 rPM; *p* = 0.033) (Figure 1A). No significant TP–FP differences were observed for HCV, CMV, HIV, or Sphinx.

Receiver operating characteristic (ROC) analysis was performed to assess the ability of blood viral burden to discriminate TP from FP cases among blood^+^ individuals. For EBV and parvovirus, ROC curves demonstrated moderate discriminative performance (EBV: AUC = 0.83; parvovirus: AUC = 0.74) (Figure 1B), with Youden-optimized sensitivity/specificity of 0.72/0.82 and 0.78/0.88, respectively.

For TTV, ROC analysis showed strong overall discrimination (AUC = 0.86), with a Youden-optimized sensitivity of 0.79 and specificity of 0.82 at a blood burden threshold of 38.5 rPM. Sufficient numbers of TP cases enabled stratification by HIV status, demonstrating that the predictive relationship between blood TTV burden and brain presence was preserved across immune contexts. Stratified ROC curves showed higher discriminative performance in HIV^+^ individuals (AUC = 0.98) compared with HIV^−^ individuals (AUC = 0.72); however, optimal Youden thresholds were similar across strata (HIV^+^: 39.31 rPM; HIV^−^: 31.32 rPM), indicating comparable operating points for prediction despite differences in discriminative strength (Figure 1C).

## 3. Discussion

In this study, we evaluated whether peripheral viral detection by unbiased mNGS, particularly in blood, could inform the presence of virus in the brain across a diverse set of viral taxa. As proof of concept, our findings suggest that blood viral detection may be informative about brain involvement for certain viruses, most commonly through rule-out utility and, in this cohort, in select cases through enrichment of brain positivity at higher blood viral burden. These findings motivate larger, systematic studies to determine whether, and for which viral taxa, blood-based virome surveillance can reliably inform CNS viral presence. This question is particularly relevant in the context of chronic CNS disease, where the feasibility and justification for direct CNS sampling differ substantially from those of acute infection.

Importantly, the viral contexts examined here differ fundamentally from fulminant CNS infections such as encephalitis or meningitis. In acute settings, such as suspected JCV–associated PML, invasive testing of brain biopsy or CSF is clinically justified and typically performed as a single diagnostic intervention. In contrast, analogous approaches are neither practical nor ethical for longitudinal monitoring of chronic CNS disease, highlighting the need for scalable peripheral indicators of brain involvement. Increasing evidence links viral presence in the brain to chronic neurodegenerative and neuroinflammatory conditions, including Alzheimer’s disease, Parkinson’s disease, multiple sclerosis, and other disorders characterized by slow progression and long preclinical phases. In such settings, viral burden may be low or intermittent, and classic symptoms of acute infection are often absent. Diagnostic paradigms optimized for acute CNS infection may therefore perform poorly when applied to chronic or smoldering viral processes.

Across viral taxa, blood negativity was generally more informative for predicting the absence of virus in the brain than blood positivity for predicting viral presence, reflected by high sensitivity and negative predictive value for several viruses. This pattern should be interpreted in the context of the very limited cohort size and low event counts for many viruses, which constrain the precision of positive predictive estimates. In small cohorts, modest numbers of false positives can disproportionately attenuate PPV, whereas sensitivity and NPV are more stable when brain-positive cases are rare. Accordingly, in this cohort, absence of detectable virus in blood often corresponded to absence in the brain, while detection in blood did not uniformly imply CNS involvement. Expansion to larger and more diverse populations will be necessary to determine whether positive predictive performance improves for specific viruses.

The viral taxa detected in this cohort were predominantly viruses capable of persistent infection in human tissues. This pattern likely reflects both biological and technical factors influencing detection in archival samples. Many viruses commonly detected in human tissues, including herpesviruses and other chronic viruses, establish lifelong or long-duration infections and are highly prevalent in the population, increasing the probability of detection in autopsy-derived material. In contrast, RNA viruses associated with neuroinvasive disease, including flaviviruses and alphaviruses, typically cause acute infections with transient viremia and limited persistence outside periods of active disease. As a result, such viruses would be expected to be uncommon in a cohort not enriched for acute encephalitic presentations. Genome characteristics may also contribute to detection patterns, as larger viral genomes provide more probe targets for hybrid-capture enrichment compared with smaller RNA viral genomes.

CSF is commonly considered the most directly accessible compartment for evaluating brain infection; however, CSF viral detection showed limited predictive utility across most viral taxa examined, with the notable exception of JCV. This pattern is likely attributable to the chronic, non-fulminant nature of infections represented in this cohort. Even for JCV, CSF sensitivity is known to be limited outside periods of active disease [10]. These findings reinforce the notion that CSF negativity should not be interpreted as the absence of a viral signal in brain tissue, particularly in surveillance contexts or preclinical disease states.

From a practical standpoint, blood represents the most accessible and scalable compartment for longitudinal surveillance. The observation that blood viral burden, as measured by rPM, was associated with brain involvement for select viruses further supports its potential utility. For EBV and parvovirus, blood viral burden demonstrated acceptable discriminative performance for distinguishing true positive from false positive cases. For TTV, blood viral burden showed strong discrimination, with consistent operating thresholds across HIV^+^ and HIV^−^ individuals despite differences in discriminative strength. These findings indicate that while predictive performance may vary by host immune context, the underlying relationship between blood burden and brain presence can be preserved.

Viruses such as TTV and Sphinx1.76 are not currently targets of routine clinical testing but have been detected in brain tissue and implicated in neuroinflammatory or neurodegenerative processes [11,12,13,14,15,16]. Because TTV is highly prevalent and not routinely studied, the ability to enrich for brain involvement using blood burden could enable large-scale, longitudinal studies to map CNS detection patterns across populations and disease states, including neurodegenerative conditions in which smoldering viral processes are hypothesized to contribute to long-term pathology. Similarly, Sphinx-related episomal DNAs have been identified in human brain tissue and linked to neuroinflammatory conditions, with emerging evidence suggesting functional interactions within neural cells. In this context, a blood-based surveillance method that can predict brain involvement could facilitate large-scale studies aimed at elucidating temporal dynamics and disease associations that are otherwise difficult to study due to limited access to brain tissue.

An important advantage of unbiased mNGS-based assays is their ability to support scalable, repeated surveillance without requiring invasive CNS sampling. In contrast to diagnostic testing designed for a single virus, unbiased sequencing enables broad detection of viral taxa across longitudinally collected peripheral samples. This capability is particularly relevant for chronic CNS disease, where repeated brain biopsy or CSF sampling is impractical, ethically constrained, and unlikely to be justified in the absence of severe neurological decline. As such, untargeted mNGS provides a practical framework for generating the large-scale peripheral data needed to evaluate blood–brain viral relationships across populations and disease states.

Several limitations should be acknowledged. Brain viral detection in this study was based on a single frontal lobe sample per donor. Prior studies, including analyses from the Last Gift cohort, have demonstrated that HIV detection can vary substantially across brain regions within the same individual, with some regions testing positive while others are negative; thus, sampling bias may be a confounding factor [17]. In addition, no measures of blood–brain barrier integrity were available for these samples. Blood–brain barrier permeability can vary across individuals and may be influenced by factors such as HIV infection, antiretroviral therapy, or substance use, such as alcohol and amphetamine usage, and could therefore modify the relationship between peripheral viral detection and CNS viral presence [18]. The cohort size is very limited, and predictive values reported here are cohort-specific and influenced by underlying prevalence and immune status. Expansion to substantially larger and more diverse populations will be necessary to establish stable patterns of predictive performance and to assess variability across age, comorbidities, immune status, and disease states. Additionally, rPM is a semi-quantitative measure influenced by technical and biological factors and should not be interpreted as a direct proxy for viral titer. Nevertheless, its consistent association with brain involvement for certain viruses suggests that relative viral burden may be informative in surveillance-oriented settings.

Peripheral “blood” samples in this cohort were not uniform and included PBMCs, lymph node tissue, and whole blood, depending on specimen availability from contributing sites. These sample types differ in cellular composition and may differentially capture cell-associated versus cell-free viruses, potentially influencing detection patterns for viruses with distinct biological tropisms. In contrast, CSF specimens were processed by centrifugation to remove cells prior to analysis, which may reduce the detection of cell-associated viruses relative to cellular blood compartments. These differences introduce heterogeneity in compartment sampling that should be considered when interpreting cross-compartment comparisons. Sequencing-based detection approaches, such as ViroFind, differ from conventional PCR assays in both scope and intended application. PCR provides highly sensitive detection of predefined viral targets and remains the standard approach for confirming infection or monitoring viral burden once a virus of interest has been identified. In contrast, hybrid-capture sequencing enables simultaneous interrogation of hundreds of viral taxa without requiring prior assumptions about which viruses may be present. In the present study, sequencing-based detection was used as a discovery framework to evaluate blood–brain viral relationships across a broad set of viral taxa. Because sequencing-based read counts represent a semi-quantitative index rather than a calibrated measure of viral load, the thresholds identified here for predicting brain involvement are specific to this platform and should not be interpreted as directly transferable to PCR-based measurements. Instead, these analyses provide a proof-of-concept framework for identifying viruses for which peripheral viral burden may track CNS involvement, which could subsequently be evaluated using targeted assays in larger longitudinal cohorts.

Taken together, these findings support the feasibility of blood-based virome surveillance as a research tool for studying chronic viral involvement in the brain. While not intended to replace diagnostic testing, such approaches may complement existing methods by enabling large-scale, hypothesis-generating investigations and by informing clinical triage in settings where rapid risk stratification is needed, and invasive CNS sampling is delayed or impractical. Establishing which blood-based viral signals reliably track CNS involvement will require substantially larger studies, but doing so could improve both research efficiency and early clinical decision-making in chronic and preclinical neurological disease.

## 4. Materials and Methods

The study was approved by the Institutional Review Board of Northwestern University (STU00211556). Human tissues were obtained from the National Neuro-AIDS Tissue Consortium (NNTC). Contributing sites operate under the following IRB-approved protocols: Texas NeuroAIDS Research Center (TNRC), University of Texas Medical Branch, IRB-approved projects 98-402 and 03-195; California NeuroAIDS Tissue Network (CNTN), University of California, San Diego, IRB-approved projects 171024 and 080323; National Neurological AIDS Bank (NNAB), University of California, Los Angeles, IRB-approved project 10-000525; and Manhattan HIV Brain Bank (MHBB), Icahn School of Medicine at Mount Sinai, IRB-approved project HS11-00388. All specimens provided to the investigators were archival and de-identified and separate informed consent was not required at Northwestern University.

### 4.1. Virome Sequencing and Viral Detection

Unbiased metagenomic virome sequencing was performed using the ViroFind platform as previously described [9,19]. Briefly, total nucleic acid was extracted from brain (frontal cortex), blood-associated samples (PBMC, lymph node, and/or spleen; collectively referred to as “blood” throughout), and CSF, followed by library preparation, hybrid capture with biotinylated RNA probes against the entire genome of >560 viruses, and 150 bp paired-end dual-indexed sequencing on the NovaSeq platform (Illumina, Inc.; San Diego, CA, USA). Viral reads were identified using a database of all viral sequences deposited in NCBI GenBank (https://www.ncbi.nlm.nih.gov/genbank/ accessed 1 September 2021), and viral detection was defined by the presence of virus-mapped reads exceeding a threshold of 10 total reads.

Quantitative analysis of viral abundance in blood was summarized as reads per million (rPM), calculated as the number of virus-mapped reads normalized to the total sequencing depth in millions of reads.

### 4.2. Qualitative Predictive Utility

For each virus in each donor, matched brain-blood and brain-CSF detection status was classified as positive or negative. Using brain detection as the outcome, blood and CSF samples were labeled as true positive (TP; virus detected in both brain and blood/CSF), false positive (FP; virus detected in blood/CSF but not in brain), true negative (TN; not detected in brain and also not detected in blood/CSF) and false negative (FN; virus detected in brain but not detected in blood/CSF). Predictive utility metrics were calculated using standard definitions: positive predictive value (PPV = TP/[TP + FP]), sensitivity (TP/[TP + FN]), negative predictive value (NPV = TN/[TN + FN]), and specificity (TN/[TN + FP]), as previously described [20]. Viruses were annotated according to predefined predictive utility categories based on PPV, specificity, NPV, and sensitivity. Rule-in utility was defined as high (≥0.9), moderate (0.8–<0.9) or poor (<0.8) for both PPV and Specificity for the ability of peripheral positivity to identify brain involvement. Rule-out utility was defined as high (≥0.9), moderate (0.8–<0.9) or poor (<0.8) for both NPV and Sensitivity for the ability of peripheral negativity to exclude brain involvement. Viruses that exhibited inter-analysis prevalence of less than 10% were not annotated. Predictive annotations were intended to describe cohort-specific performance rather than establish diagnostic classification.

### 4.3. Quantitative Predictive Utility

Blood viral-reads were quantified into blood burden in rPM, which were compared between TP and FP using two-sided Wilcoxon rank-sum tests. Significant differences were plotted on jittered dot plots using ggplot2 (v 3.52; Posit Software, PBC, Boston, MA, USA) [21] for R (R Foundation for Statistical Computing, Vienna, Austria)/R-studio (v 2024.12.1+563; Posit Software, PBC, Boston, MA, USA) on a log10(rPM + 1) scale and were further analyzed for the Youden index and receiver operator characteristic (ROC) analysis.

ROC curves were constructed using blood rPM as a continuous predictor and brain presence as a binary outcome using pROC (v 1.19.0.1) for R/R-studio [22]. For each ROC curve, the optimal operating point was determined using the Youden index, defined as sensitivity + specificity − 1. The Youden-optimized threshold, sensitivity, and specificity were extracted for each virus. For TTV, Youden thresholds were calculated for the combined cohort and separately for HIV^+^ and HIV^−^ individuals. Stratified Youden analyses were not emphasized for EBV or parvovirus due to insufficient numbers of TP cases in HIV^−^ individuals. Area under the curve (AUC) was calculated as a measure of discriminative performance. ROC plots display sensitivity versus 1—specificity, with diagonal reference lines indicating no discrimination. In-house R script available by request.

## Figures and Tables

**Table 1:** Predictive utility of virus detection in blood for identifying viral presence in the brain.

Virus	TP	FP	FN	TN	PPV	Specificity	Rule-In	NPV	Sensitivity	Rule-Out	Utility
EBV	18	38	1	9	0.32	0.19	Poor	0.9	0.95	High	Rule-out
HHV7	1	41	0	24	0.02	0.37	Poor	1	1	High	Rule-out
HIV	12	15	1	38	0.44	0.72	Poor	0.97	0.92	High	Rule-out
HCV	4	7	0	55	0.36	0.89	Poor	1	1	High	Rule-out
TTV	33	31	1	1	0.52	0.03	Poor	0.5	0.97	Poor	None
CMV	31	8	14	13	0.79	0.62	Poor	0.48	0.69	Poor	None
HHV6 A/B	5	33	3	25	0.13	0.43	Poor	0.89	0.63	Poor	None
Parvovirus	9	25	6	26	0.26	0.51	Poor	0.81	0.6	Poor	None
Adenovirus	0	0	26	40	N/A	1	N/A	0.61	0	Poor	None
Sphinx1.76	6	3	14	43	0.67	0.93	Poor	0.75	0.3	Poor	None
Pegivirus	1	2	11	52	0.33	0.96	Poor	0.83	0.08	Poor	None
JCV	0	0	11	55	N/A	1	N/A	0.83	0	Poor	None
Papillomavirus	1	3	7	55	0.25	0.95	Poor	0.89	0.13	Poor	None
HSV1	2	3	2	59	0.4	0.95	Poor	0.97	0.5	Poor	N/A
AAV	2	0	4	60	1	1	High	0.94	0.33	Poor	N/A
HBV	3	0	3	60	1	1	High	0.95	0.5	Poor	N/A
HHV8	0	4	0	62	0	0.94	Poor	1	N/A	N/A	N/A
HSV2	2	0	1	63	1	1	High	0.98	0.67	Poor	N/A
BKV	1	1	1	63	0.5	0.98	Poor	0.98	0.5	Poor	N/A
MCV	0	3	0	63	0	0.95	Poor	1	N/A	N/A	N/A
Coronavirus	1	1	0	64	0.5	0.98	Poor	1	1	High	N/A
VZV	0	2	0	64	0	0.97	Poor	1	N/A	N/A	N/A
Human parainfluenza	0	2	0	64	0	0.97	Poor	1	N/A	N/A	N/A
HPyV6	0	2	0	64	0	0.97	Poor	1	N/A	N/A	N/A
WU Virus	1	0	0	65	1	1	High	1	1	High	N/A
HTLV 2	1	0	0	65	1	1	High	1	1	High	N/A
SV40	0	0	1	65	N/A	1	N/A	0.98	0	Poor	N/A
Norovirus	0	1	0	65	0	0.98	Poor	1	N/A	N/A	N/A
RSV	0	1	0	65	0	0.98	Poor	1	N/A	N/A	N/A

TP: True positive; FP: False positive; FN: False negative; TN: True negative; PPV: Positive Predictive Value; NPV: Negative Predictive Value; N/A: not applicable (gray text).

**Table 2:** Predictive utility of virus detection in CSF for identifying viral presence in the brain.

Virus	TP	FP	FN	TN	PPV	Specificity	Rule-In	NPV	Sensitivity	Rule-Out	Utility
JCV	2	0	7	15	1	1	High	0.68	0.22	Poor	Rule-In
HBV	3	5	0	16	0.38	0.76	Poor	1	1	High	Rule-out
TTV	9	6	7	2	0.6	0.25	Poor	0.22	0.56	Poor	None
CMV	2	3	15	4	0.4	0.57	Poor	0.21	0.12	Poor	None
Adenovirus	0	0	16	8	N/A	1	N/A	0.33	0	Poor	None
HIV	4	4	5	11	0.5	0.73	Poor	0.69	0.44	Poor	None
EBV	4	2	6	12	0.67	0.86	Poor	0.67	0.4	Poor	None
Sphinx1.76	1	2	9	12	0.33	0.86	Poor	0.57	0.1	Poor	None
Papillomavirus	1	6	3	14	0.14	0.7	Poor	0.82	0.25	Poor	None
Parvovirus	0	2	6	16	0	0.89	Poor	0.73	0	Poor	None
HHV6 A/B	0	1	4	19	0	0.95	Poor	0.83	0	Poor	None
AAV	0	0	3	21	N/A	1	N/A	0.88	0	Poor	None
HHV7	0	2	1	21	0	0.91	Poor	0.95	0	Poor	None
BKV	0	0	2	22	N/A	1	N/A	0.92	0	Poor	N/A
STLPyV	1	1	0	22	0.5	0.96	Poor	1	1	High	N/A
MCV	0	2	0	22	0	0.92	Poor	1	N/A	N/A	N/A
HSV-1	0	0	1	23	N/A	1	N/A	0.96	0	Poor	N/A
HSV-2	0	0	1	23	N/A	1	N/A	0.96	0	Poor	N/A
WU Virus	0	0	1	23	N/A	1	N/A	0.96	0	Poor	N/A
Pegivirus	0	0	1	23	N/A	1	N/A	0.96	0	Poor	N/A
HCV	0	1	0	23	0	0.96	Poor	1	N/A	N/A	N/A
Sewage-associated gemycircularvirus	0	1	0	23	0	0.96	Poor	1	N/A	N/A	N/A
SV40	0	1	0	23	0	0.96	Poor	1	N/A	N/A	N/A

TP: True positive; FP: False positive; FN: False negative; TN: True negative; PPV: Positive Predictive Value; NPV: Negative Predictive Value; N/A: not applicable (gray text).

**Table 3:** Predictive utility of virus detection in blood for identifying viral presence in the brain of HIV positive people.

Virus	TP	FP	FN	TN	PPV	Specificity	Rule-In	NPV	Sensitivity	Rule-Out	Utility
HBV	3	0	2	31	1	1	High	0.94	0.6	Poor	Rule-in
TTV	19	16	0	1	0.54	0.06	Poor	1	1	High	Rule-out
EBV	17	18	0	1	0.49	0.05	Poor	1	1	High	Rule-out
HIV	12	15	0	9	0.44	0.38	Poor	1	1	High	Rule-out
HHV7	1	19	0	16	0.05	0.46	Poor	1	1	High	Rule-out
HCV	4	4	0	28	0.5	0.88	Poor	1	1	High	Rule-out
CMV	22	5	4	5	0.81	0.5	Poor	0.56	0.85	Poor	None
Parvovirus	7	14	4	11	0.33	0.44	Poor	0.73	0.64	Poor	None
Adenovirus	1	1	21	13	0.5	0.93	Poor	0.38	0.05	Poor	None
HHV6 A/B	1	17	3	15	0.06	0.47	Poor	0.83	0.25	Poor	None
Sphinx1.76	3	3	8	22	0.5	0.88	Poor	0.73	0.27	Poor	None
Pegivirus	1	2	6	27	0.33	0.93	Poor	0.82	0.14	Poor	None
JCV	0	8	0	28	0	0.78	Poor	1	N/A	N/A	None
Papillomavirus	1	1	5	29	0.5	0.97	Poor	0.85	0.17	Poor	None
AAV	0	1	3	32	0	0.97	Poor	0.91	0	Poor	None
HHV8	0	4	0	32	0	0.89	Poor	1	N/A	N/A	None
HSV2	2	0	1	33	1	1	High	0.97	0.67	Poor	N/A
HSV1	0	2	1	33	0	0.94	Poor	0.97	0	Poor	N/A
MCV	0	2	0	34	0	0.94	Poor	1	N/A	N/A	N/A
HPyV6	0	2	0	34	0	0.94	Poor	1	N/A	N/A	N/A
BKV	1	0	0	35	1	1	High	1	1	High	N/A
VZV	1	0	0	35	1	1	High	1	1	High	N/A
WU Virus	1	0	0	35	1	1	High	1	1	High	N/A
Human parainfluenza	0	1	0	35	0	0.97	Poor	1	N/A	N/A	N/A
Norovirus	0	1	0	35	0	0.97	Poor	1	N/A	N/A	N/A
RSV	0	1	0	35	0	0.97	Poor	1	N/A	N/A	N/A
SV40	0	1	0	35	0	0.97	Poor	1	N/A	N/A	N/A

TP: True positive; FP: False positive; FN: False negative; TN: True negative; PPV: Positive Predictive Value; NPV: Negative Predictive Value; N/A: not applicable (gray text).

**Table 4:** Predictive utility of virus detection in blood for identifying viral presence in the brain of HIV negative people.

Virus	TP	FP	FN	TN	PPV	Specificity	Rule-In	NPV	Sensitivity	Rule-Out	Utility
Sphinx1.76	3	0	5	22	1	1	High	0.81	0.38	Poor	Rule-in
AAV	1	0	2	27	1	1	High	0.93	0.33	Poor	Rule-in
EBV	1	20	0	9	0.05	0.31	Poor	1	1	High	Rule-out
HHV6 A/B	4	16	0	10	0.2	0.38	Poor	1	1	High	Rule-out
TTV	14	15	1	0	0.48	0	Poor	0	0.93	Poor	None
CMV	9	3	10	8	0.75	0.73	Poor	0.44	0.47	Poor	None
HHV7	0	22	0	8	0	0.27	Poor	1	N/A	N/A	None
Parvovirus	2	11	2	15	0.15	0.58	Poor	0.88	0.5	Poor	None
Adenovirus	0	0	5	25	N/A	1	N/A	0.83	0	Poor	None
Pegivirus	0	0	5	25	N/A	1	N/A	0.83	0	Poor	None
HSV1	2	1	1	26	0.67	0.96	Poor	0.96	0.67	Poor	None
Papillomavirus	0	2	2	26	0	0.93	Poor	0.93	0	Poor	None
HCV	0	3	0	27	0	0.9	Poor	1	N/A	N/A	None
JCV	0	3	0	27	0	0.9	Poor	1	N/A	N/A	None
BKV	0	1	1	28	0	0.97	Poor	0.97	0	Poor	N/A
Coronavirus	1	1	0	28	0.5	0.97	Poor	1	1	High	N/A
HBV	0	0	1	29	N/A	1	N/A	0.97	0	Poor	N/A
HTLV 2	1	0	0	29	1	1	High	1	1	High	N/A
MCV	0	1	0	29	0	0.97	Poor	1	N/A	N/A	N/A
Human parainfluenza	0	1	0	29	0	0.97	Poor	1	N/A	N/A	N/A

TP: True positive; FP: False positive; FN: False negative; TN: True negative; PPV: Positive Predictive Value; NPV: Negative Predictive Value; N/A: not applicable (gray text).

## Data Availability

The original contributions presented in this study are included in the article/Supplementary Materials. Further inquiries can be directed to the corresponding author.

## Supplementary Materials

The following supporting information can be downloaded at: https://www.mdpi.com/article/10.3390/ijms27062789/s1.
