# Supplementary material for: Predictive Utility of ViroFind Detection of Blood and CSF Virome for Viral Presence in Human Brain Tissue
Source: Int J Mol Sci. 2026 Mar 19;27(6):2789. doi: 10.3390/ijms27062789 (PMC13026825; doi:10.3390/ijms27062789)
Supplement: Supplementary file 1 [file ijms-27-02789-s001.zip › ijms-4190892-supplementary.pdf]

Supplementary Table S1. Full names of viruses referenced in this study

Abbreviation	Full Virus Name	Family	Genome Type
AAV	Adeno-associated virus	Dependoparvovirus	ssDNA
Adenovirus	Human adenovirus	Adenoviridae	dsDNA
BKV	BK polyomavirus	Polyomaviridae	dsDNA
CMV	Human cytomegalovirus	Herpesviridae	dsDNA
Coronavirus	Human coronaviruses	Coronaviridae	ssRNA(+)
EBV	Epstein–Barr virus	Herpesviridae	dsDNA
HBV	Hepatitis B virus	Hepadnaviridae	partially dsDNA
HCV	Hepatitis C virus	Flaviviridae	ssRNA(+)
HHV6 A/B	Human herpesvirus 6A / 6B	Herpesviridae	dsDNA
HHV7	Human herpesvirus 7	Herpesviridae	dsDNA
HHV8	Human herpesvirus 8 (Kaposi sarcoma–associated herpesvirus)	Herpesviridae	dsDNA
HIV	Human immunodeficiency virus	Retroviridae	ssRNA(+)/dsDNA
HPyV6	Human polyomavirus 6	Polyomaviridae	dsDNA
HSV1	Herpes simplex virus type 1	Herpesviridae	dsDNA
HSV2	Herpes simplex virus type 2	Herpesviridae	dsDNA
HTLV 2	Human T-lymphotropic virus type 2	Retroviridae	ssRNA(+)/dsDNA
Human parainfluenza	Human parainfluenza virus	Paramyxoviridae	ssRNA(−)
JCV	JC polyomavirus	Polyomaviridae	dsDNA
MCV	Merkel cell polyomavirus	Polyomaviridae	dsDNA
Norovirus	Human norovirus	Caliciviridae	ssRNA(+)
Papillomavirus	Human papillomavirus	Papillomaviridae	dsDNA
Parvovirus	Human parvovirus B19	Parvoviridae	ssDNA
Pegivirus	Human pegivirus (GB virus C)	Flaviviridae	ssRNA(+)
RSV	Respiratory syncytial virus	Pneumoviridae	ssRNA(−)
Sewage-associated gemycircularvirus	Sewage-associated gemycircularvirus	Genomoviridae	ssDNA
Sphinx1.76	Sphinx 1.76 virus	Genomoviridae	ssDNA
STLPyV	Saint Louis polyomavirus	Polyomaviridae	dsDNA
SV40	Simian virus 40	Polyomaviridae	dsDNA
TTV	Torque teno virus	Anelloviridae	ssDNA
VZV	Varicella-zoster virus	Herpesviridae	dsDNA
WU Virus	WU polyomavirus	Polyomaviridae	dsDNA
